# A Routing Algorithm Based on Real-Time Information Traffic in Sparse Environment for VANETs

**DOI:** 10.3390/s20247018

**Published:** 2020-12-08

**Authors:** Jianhang Liu, Fan Bai, Haonan Weng, Shibao Li, Xuerong Cui, Yucheng Zhang

**Affiliations:** 1 College of Computer Science and Technology, China University of Petroleum (East China), Qingdao 266580, China; 2 College of Oceanography and Space Informatics, China University of Petroleum (East China), Qingdao 266580, China; S18070001@s.upc.edu.cn (F.B.); 1507030116@s.upc.edu.cn (H.W.); Lishibao@upc.edu (S.L.); cur@upc.edu.cn (X.C.); 3 CAS Engineering Laboratory for Intelligent Agricultural Machinery Equipment, Beijing 100000, China; zyc@ict.ac.cn

**Keywords:** routing protocol, vehicular ad hoc networks, connectivity, information traffic

## Abstract

Because of the specific characteristics, like high vehicular mobility, unstable topology, and interruption of inter-vehicle wireless communication, it is hard to make a perfect decision on packet forwarding in highly dynamic topology VANETs. Especially in a sparse urban environment, the poor connectivity of nodes will mostly cause problems such as data packet loss and routing redundancy. Therefore, how to choose the best relay node becomes a key challenge in the design of fast and reliable routing protocols. This paper presents real-time effective information traffic routing (RTEIT), which provides an optimal route for forwarding the data packets toward their destination when choosing the relay node. RTEIT introduces a new network parameter named effective information traffic which can estimate the connectivity of nodes by the path that has been successfully created. Moreover, for avoiding unexpected communication interruption, we propose a new formula to evaluate the status of the links via considering the speed, direction, and location information. Finally, the node utility, as the criterion of routing decision, is calculated by effective information traffic and link status. We use the simulator of SUMO and NS-3 platform to evaluate RTEIT, and the results are compared with GPSR MOPR, and MM-GPSR. The evaluation results demonstrate that RTEIT outperforms in terms of packet loss rate, end-to-end delay, and network yield.

## 1. Introduction

Over the past few decades, due to the fast development of the social economy and wireless communication technology, the existing road infrastructure and equipment have been unable to meet the safety and entertainment requirements of vehicles. As the next generation of the transportation system, intelligent transportation system (ITS) undertakes the task of improving road congestion, reducing traffic accidents and providing entertainment services. Vehicular Ad Hoc Networks (VANETs) are the keystone for enhancing roads users’ safety and comfort [1,2,3,4,5]. The concept of VANETs comes from the mobile ad hoc network (MANETs), VANETs combines wireless communication with the vehicle driving scenarios to enable the mutual communication between vehicle nodes [6,7,8,9]. Although there are many similarities between MANETs and VANETs, the specific characteristics of the vehicle motion model make VANETs have more problems and challenges [10,11]. For instance, the high mobility of vehicles makes frequent changes in the topology of network in VANETs, resulting in difficulties in routing table maintenance and poor node connectivity. The vehicle can only drive in one direction and may meet traffic jams or emergency accidents, these conditions will cause the communication status between vehicle nodes to be extremely complicated.

For decades, many researchers have made their own contributions in the design of routing protocol for VANETs and proposed different routing algorithms [12,13,14,15,16,17]. Some of them are classified as topology-based, cluster-based, position-based, broadcast-based, and infrastructure-based. By comparison, location-based routing represents a promising scheme in vehicular environments as it supports geographical position information of each vehicle to offer routing. When a source node starts to send packets to the destination node, the route path has not been determined because the relay node is calculated from the location information of itself, neighbors, and the destination. Nodes neither maintains the routing table nor establishes the global path between source and destination. This routing scheme can better adapt to frequent topology changes. However, these location-based routing protocols have certain limitations. The connectivity of the nodes is not taken into account in the routing algorithm, which is magnified in sparse scenarios. To overcome this problem, many new routing algorithms have been proposed. Some of them use Road Side Units (RSU) to make statistics on the road conditions, such as traffic volume, road connectivity, etc. Although RSUs can be used to solve such problems and facilitate communication among vehicles, the deployment of these units entails a high cost. Another solution is to use maps to obtain road and intersection information. There are already some commercial maps available, such as Google, Ali, and Tencent. However, commercial maps cannot provide excellent real-time performance. In some places, the network connection of these maps will be disconnected, and sometimes the latest map information may not be updated for various reasons. The other protocols that consume network resources and obtain information on vehicular density generate additional overhead.

On the other hand, in the transmission process of the current node and relay node, packet loss often occurs due to node mobility and channel loss. For example, the receiver may leave the wireless communication transmission range of the sender before receiving the packets totally.

Therefore, appropriate routing should consider both packet loss and node connectivity in the decision-making process. In this paper, we propose a relay selection algorithm exploiting hello message exchanged between vehicles, and combining several parameters in order to improve the packet loss rate and end-to-end delay performance in sparse scenarios. A new network parameter named effective information traffic is proposed in this paper which is used to assist routing decisions for reducing routing redundancy. Since the analysis of node mobility model, we propose link Utility to reduce the packet loss rate.

The main contributions of this paper are as follows:

1. We propose a link utility algorithm in VANETs, which is used to minimize the number of hops while ensuring communication quality. In this algorithm, both the vehicle’s speed, direction, and position are taken into account.
2. We propose an effective information traffic algorithm in VANETs, which is used to count the effective historical transmission information of each node, and indicate the connectivity of the node. In the algorithm, we define a function that can automatically attenuate to adapt to the change of connectivity caused by vehicle movement.
3. Based on 1 and 2, we propose a weight-based vehicle’s utility algorithm, and the weights are the variances of effective information traffic and link utility. In the relay selection process, the node with the highest vehicle utility is selected as the relay node.

The remaining of this paper is organized as follows: Previous related studies are reviewed in Section 2. The effective information traffic is described in Section 3. Unveiling the modules and the main algorithms of our relay selection scheme is the subject of Section 4. Section 5 presents the simulation and analysis of the experimental results. Finally, Section 6 concludes our work in this paper and points out the further works.

## 2. Related Work

### 2.1. Topology-Based Routing Protocols

Many researchers regard topology as the basic element of routing protocols and design some routing protocols based on topology. In topology-based routing protocols, each node maintains a routing table for forwarding packets from source to destination. For Destination Sequenced Distance Vector Routing (DSDV) [18], each node in the network topology maintains the topology information about all the other nodes, and exchange information with neighbors at fixed time intervals to ensure the validity of topology information. The discovery and maintenance of routing tables are proactive, based on time intervals, and using broadcasts. When a message needs to be sent, the source node can quickly find the routing path from the routing table, and then quickly forward the packet to the destination node. This scheme provides a relatively low network delay but results in substantial network overhead in the process of exchanging information while the network scale is large. Ad-hoc On-Demand Distance Vector (AODV) [19] is a reactive routing protocol, which establishes the path only after the node wishes to engage in communication. This lightens the burden on the network. In route discovery, the source node sends packets to the destination node by flooding. The relay nodes forward the packet until the destination node receives it. In the whole routing discovery process of AODV routing, a lot of network overhead is generated.

### 2.2. Clustering-Based Routing Protocol

In order to improve the performance of routing protocols, some scholars propose clustering-based routing protocols. Cluster is a kind of network technology that groups a large number of nodes into sub-networks. On the one hand, clustering can better adapt to the VANET environment, on the other hand, clustering is easy to manage the nodes in the cluster, and has certain advantages in routing selection, bandwidth allocation, and channel access. There are two hotspots in cluster-based routing protocol research: firstly, how to cluster the vehicle nodes more effectively; secondly, how to construct reliable and low delay routing links by clustering. Chen et al. [20] design a clustering algorithm according to the distance and density of nodes. Due to the inherent characteristics of continuous change of moving object position, clustering results will change dynamically. By analyzing the characteristics of urban street network, the concept of cluster block (CB) is introduced as the bottom cluster unit. In order to achieve high-quality multimedia transmission, Tal et al. [21] propose a user-oriented VANET multimedia transmission solution based on clustering, which can transmit multimedia content according to passengers’ preferences. Merely discussing how to form more effective clusters does not ultimately solve the data transmission problem in VANET. In addition, some researchers have studied how to build routes. Kayis et al. [22] propose a stable routing method based on passive clustering. Considering the mobility of nodes, this clustering method only assigns tasks to vehicles that need to communicate. Due to the characteristics of high-speed motion of nodes in VANET, Lin et al. [23] propose a clustering method based on moving motion regions. When forwarding data, a route is constructed through cluster edge nodes to surrounding cluster head nodes.

### 2.3. Position-Based Routing Protocols

GPSR is one of the most classic and famous routing protocols in location-based routing protocols [24,25,26]. It uses GPS positioning system of vehicle equipment for routing. Each node stores the location information of adjacent nodes, and links are created directly through the location information. It makes use of two forwarding schemes for sending packets: perimeter mode (recovery mode) and greedy mode. In greedy forwarding, the sender selects the vehicle which is nearest to the destination in neighbors as the receiver according to the location of the destination and the neighbors. Periodically, the nodes exchange this information with their one-hop neighbors through hello messages. If the greedy mode fails, the current node enters the peripheral forwarding that uses the right-hand rule. GPCR [27], based on the GPSR protocol, has made relevant improvements for intersections on the internet of vehicles. GPCR divides greedy forwarding into two cases. While the next hop is in the current road segment, the node closest to the destination node is selected as the next hop. On the contrary, the node at the intersection is selected as the next-hop node. Yang et al. have proposed MM-GPSR [28], based on the maximum transmission time and minimum angle. By quantifying the transmission time, the vehicle with the longest communication time is selected for the greedy forwarding, and the vehicle with the smallest angle is used to forward when the topology hole is encountered. It improves the link quality of greedy forwarding communication and reduces the redundancy of the right-hand rule in peripheral forwarding transmission. Connectivity aware routing protocol (CAR) [29] is a kind of routing scheme based on connectivity awareness. The source node finds the path information of the destination node through broadcast. In order to achieve good connectivity and link quality, the number of hops and the number of neighbor nodes will be updated to the packet in each forwarding process. After receiving, the destination node selects and responds to the source node. Although CAR solves the problem of connection, the accuracy of information along the way cannot be guaranteed, and the topology will change.

## 3. Effective Information Traffic

### 3.1. Node Connectivity

**Figure 1:**
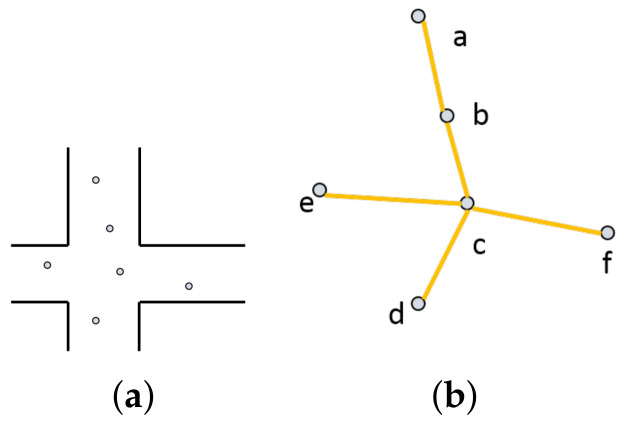
Topological network: (**a**) actual environment; (**b**) micro topology.

In the general urban network environment, only the road section in the center of the city has high vehicle density most of the time, which can provide better communication conditions for the network. In most cases, the vehicle density is unsaturated. In the sparse urban environment, the number of vehicles is far from enough to make each vehicle can communicate with any node in the network. Thus, for the whole large network, the network topology can be divided into many scattered small topologies. After such an operation, it is convenient for us to analyze the network. As Figure 1 shows an example:

In Figure 1, the wireless connection between vehicles can be expressed as undirected graph $G=(V_{N},E_{M})$, where the nodes set $V_{N}$ composed of N nodes is represented as $V_{N}=\left\{v_{1},v_{2},v_{3},\ldots ,v_{N}\right\}$, and $E_{M}=\left\{e_{1},e_{2},e_{3},\ldots ,e_{M}\right\}$ denotes the set of wireless links. In this simple network, node connectivity can be defined as the following formula:

(1) $$C\left(v\right)=\sum_{s\neq v\neq t}\frac{\sigma_{st}\left(v\right)}{\sigma_{st}},$$

where $\sigma_{st}\left(v\right)$ denotes the number of shortest path through node *v* from *s* to *t*, and $\sigma_{st}$ is numbers of whole shortest path from *s* to *t*. From (b), it shows a simple topology, each path is the shortest path, which is easy for us to analyze. In the nodes set $V=\left\{a,e,d,f\right\}$, each node is a node at the edge, and there is no shortest path through it, at the same time they have the worst connectivity. In contrast, node c is the node at the center of the topology while also has the highest C(v). In Figure 1, the absence of nodes in the graph indicates that there is no available route at that time. For node *b*, the candidate set of destination nodes is divided into two cases, in the micro topology and not in the micro topology. When the destination node is not in the micro topology, the selection of routing nodes has little effect on whether the destination node can reach the destination node because the topology changes can not be predicted. If the destination node is in micro topology, node *c* is the best relay node for node *b*. Because if node *c* is disconnected from *b*, the number of reachable nodes for node *b* will be reduced. Therefore, it is necessary to consider the connectivity of nodes in routing. However, due to the limitation of the actual situation, we can not get all the topological paths in real-time, so we introduce effective information traffic.

### 3.2. Effective Information Traffic

**Figure 2:**
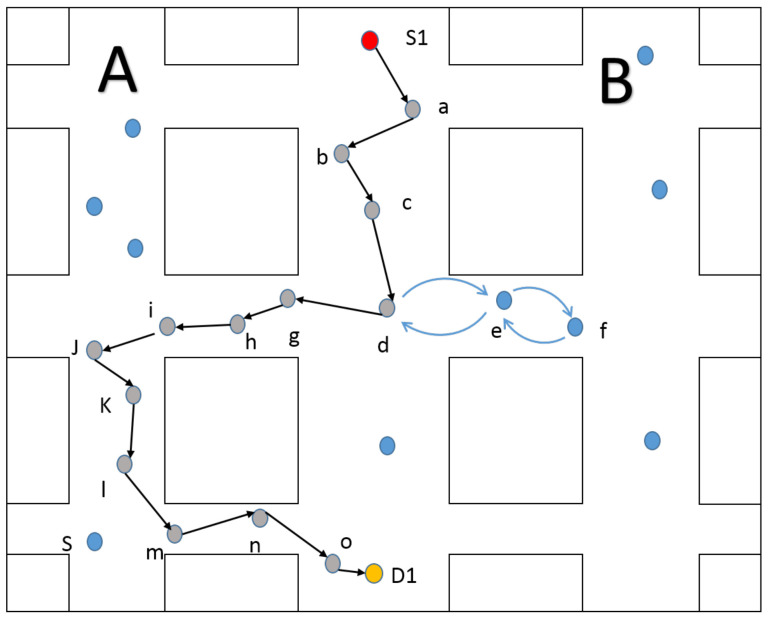
Forwarding example.

We will use an example in Figure 2 to illustrate our motivation to introduce effective information traffic.

Consider Figure 2, where node S1 intends to send packet to node D1. It is clear that there is an optimal routing path through node *d*, $P\;=\;\left\{S1,a,b,c,d,g,h,i,j,k,l,m,n,o,D1\right\}$. This routing path will provide the best network performance, such as end-to-end delay and delivery ratio. In GPSR, when the packet is sent to node *d*, it will calculate the distance between neighbors and D1 to select the nearest node to the D1. Since delay selection only depends on the location information, it mostly tends to select node *e* as the next hop which leads the packet to the topology hole. The routing path will be created as $P\;=\;\left\{e,f,e,d\right\}$ after node d send packet to *e* in GPSR mode. Redundant path $P\;=\;\left\{e,f,e,d\right\}$ increases the number of hops which is the main factor affecting end-to-end delay. Additionally, a higher number of hops will increase the packet loss rate while topology changes due to the mobility of vehicles. The traditional solution is traffic-aware which uses RSU or broadcast to get the vehicle density of the road segment and then select the segment with higher vehicle density for transmission. However, there are two drawbacks in this approach: (1) additional network consumption, like RSU device and overhead for broadcast; (2) it is not suitable for a sparse network to express the link degree by vehicle density. Because it is very likely that the vehicles will gather together and cause the node density of this road high while the network hole still exists. In some cases, the same vehicle density may lead to different connectivity results.

In this paper, we introduce a novel concept called effective information traffic to solve this problem. By analyzing the node connectivity of Formula (1) and the simple instance in Figure 2, we can get the conclusion that the central nodes have better connectivity than edge nodes. From Figure 2, routing redundancy can be avoided by using the central nodes as the relay node, such as node *g*. However, in practical application, we can not use the node connectivity defined by Formula (1) to compare which is the best relay node in the neighbors. The reason is that the global topology is unknowable. Each forwarding node can only get the location of the current node and destination node. If the method of the broadcast is used to obtain the location and connection information of all nodes, there will be a lot of network resource consumption. It is unwise to design a protocol that uses this method because of the frequent topology changes.

Therefore, we propose to use effective information traffic to replace the node connectivity defined in (1). The effective information traffic can be described as: the amount of data effectively forwarded by a node in a certain time *t*. Effectively forwarding denotes a successful forwarding process including receiving and sending to the best relay node. Correspondingly, invalid information traffic is described as the information traffic in recovery mode. For instance, while routing path $P\;=\;\left\{S1,a,b,c,d,g,h,I,j,k,l,m,n,o,D1\right\}$ is creating, all of the nodes in the path get a higher effective information traffic which leads to the situation that packet is sent to the central nodes rather than edge nodes. If the packet is sent to edge nodes like node e, the forwarding mode mostly inter to recovery mode at the node of *f* because there is no node closer than node *f* to the destination. In this situation, although the effective information traffic of nodes in path P=[d,e,f] increase, the transmission process on the path p=[f,e,d] is considered as invalid transmission, which will take time but can not let EIT increases. In this mechanism, the node with high EIT will be more competitive in the relay selection.

Due to the fact that nodes in the road are moving, the changes of topology structure will lead to the EIT can not reflect the connectivity of nodes in real-time. Therefore, we define the self attenuation function to correct the topology changes caused by motion. In the following part, we will explain the mathematical formula and maintenance method of effective information traffic.

All vehicles can calculate their effective information traffic, and add it to the hello message which is sent to neighbors periodically. Effective information traffic is defined on the basis of information flow (2).

(2) $$I\left(t\right)=\frac{P}{t},$$

where *P* is the amount of information contained in the packet for effective forwarding, and *t* is the time taken to send data *P*. In our protocol, the packets forwarded in greedy mode become valid packets and enter into the calculation of effective information traffic, while packets forwarded in recovery mode are called invalid packets which are not included in EIT calculation. The information of forwarding mode is added to the hello message and periodically forwarded to neighbor nodes.

The value of I(t) of the central node increases faster than that of the edge node, because of the preference of the central node in routing. Such a positive feedback mechanism will lead to the problem of excessive preference. The nodes which are originally located in the center have a higher increasing rate of the value of I(t). However, when the node moves from the center to the edge, the excessive preference makes the I(t) value too large to reflect the connectivity of the node correctly. Therefore, we define the attenuation function to reduce the influence of excessive preference. Each node maintains its own I(t), so the attenuation function is also performed by each node. After sending the hello packet, the sender executed the function periodically.

When the vehicle is stationary, it can be considered that the information flow can correctly reflect the node connectivity. Because the vehicle is moving, the change in the location of the vehicle will cause the node connectivity to change. Therefore, we introduce the self decay function to adapt to changes in topology. Previous valid forwarding data does not correctly reflect the current node connectivity, so the EIT needs self-reduction to ensure timeliness. We define the half-life time $t_{h}$. In other words, if the node does not make effective transmission in time $t_{h}$, EIT will become half of the original. For example, the initial information traffic is $\sigma_{0}$, and there is no effective data transmission from 0 to $t_{h}$, then at $t_{h}$ the EIT is $1/2\sigma_{0}$. Attenuation function can be obtained as below:

(3) $$I\left(t\right)=\left\{\begin{array}{c} \partial_{0}{\left(\frac{1}{2}\right)}^{\left(\frac{t}{t_{h}}\right)},t\in \;\left[0,T_{s}\right)\\ 0,t\in \;\left[T_{s},+\infty \right) \end{array}\right.$$

If a node does not effectively forward for a long time, we consider that the connectivity of the node is zero. Obviously, no matter how long the time has passed, the value of the function cannot be zero. Therefore, we introduce reset time $T_{s}=2t_{h}$. If the node does not effectively forward within $T_{s}$, the I(t) of the node will be reset. Morever, if node sends packets during the period of $T_{s}$, the time to reset I(t) will be recalculated. For example, during $\left[t_{0}-t_{1}\right]$, node does not forward effectively, so I(t) decreases as (3). At the time of $t_{1}$, the node begins to forward packet in greedy mode and the ending of transmission at $t_{2}$. If node has no transmission requirement from $t_{2}$, the reset time of I(t) is $t_{2}+T_{s}$. The value of $I(t_{2})$ is the sum of the original decay rate up to the value of $t_{2}$ and the value of σ from $t_{1}$ to $t_{2}$, as (4).

(4) $$I\left(t\right)=\left\{\begin{array}{c} \partial_{0}{\left(\frac{1}{2}\right)}^{\left(\frac{t}{t_{h}}\right)}+\frac{P}{t},t\in \;\left[0,T_{s}\right)\\ \frac{P}{t},t\in \;\left[T_{s},+\infty \right) \end{array}\right.$$

In fact, the node does not need to calculate the current I(t) in real-time and does not need to update in real-time. Just recalculate the value of I(t) and add it to the hello packet before sending it. Therefore, it is required to recalculate the I(t) with $t_{h}$ as cycle. In this paper, we set the reset interval as twice as the hello packet interval: $T_{s}=2T_{h}=2t_{h}$. Under such a mechanism, I(t) can reflect the connectivity of a node and be considered in routing decisions.

**Figure 3:**
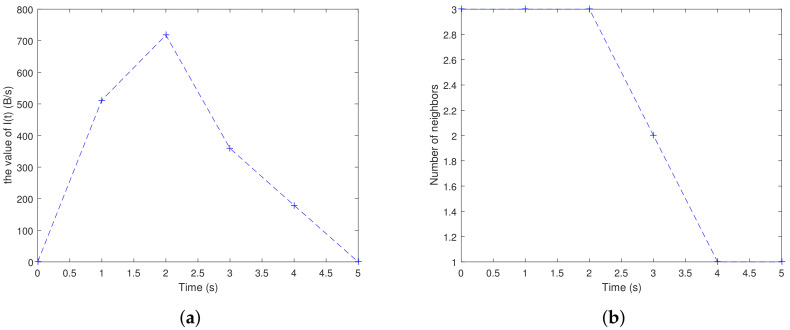
Parameters of the node: (**a**) effective information traffic; (**b**) number of neighbors.

Figure 3 shows the variety of effective information traffic and number of neighbors for a node in a period of time. The node gradually moves from the center area to the edge area. It can be found that at the time of 0 s, the number of neighbors is 3, but there is no data is forwarded to the node, and the effective information flow is 0. The value of *I*(*t*) is increased after forwarding at 1 s and 2 s, with the number of neighbors is 3. At 3 s, 4 s, and 5 s, the *I*(*t*) decreased to 0.

## 4. Routing Algorithm

It is a challenging task to design a fast and reliable routing protocol for VANETs especially in sparse urban scenes where the connection between vehicle nodes is not enough, while several aspects need to be considered to improve the routing performance.

Our contribution aims at: (i) increasing the probability of successful transmission within the communication range, (ii) increasing the probability of establishing an effective route, (iii) selecting the bests relay with the lowest delay and the highest probability of reach destinations. To achieve these goals, we define link utility and node effective information traffic and use a variance-based weight calculation method to calculate vehicle utility. The node with the largest vehicle utility will be selected as the next hop.

### 4.1. Link Utility

**Figure 4:**
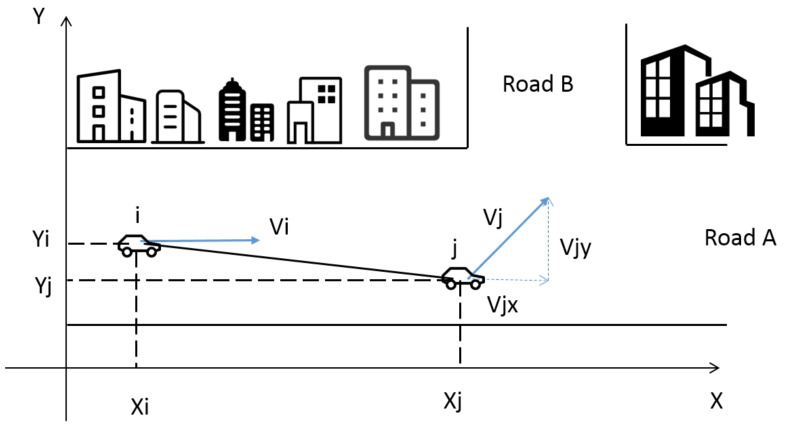
Node movement model.

Vehicle movement model can be described by the following variables: current position at $t_{0}$ in Cartesian coordinates: $X(t_{0})$ and $Y(t_{0})$; velocity vector: $\left(V_{ix}\left(t_{0}\right),V_{iy}\left(t_{0}\right)\right)$(Figure 4).

The euclidean distance between node *i* and *j* at $t_{0}$: $d_{ij,t_{0}}$ is written as (5). $x_{i,t_{0}}$ and $x_{j,t_{0}}$ are the coordinates of the x-axis of node *i* and *j* at $t_{0}$, $y_{i,t_{0}}$ and $y_{j,t_{0}}$ are the coordinates of the y-axis of node *i* and *j* at $t_{0}$. At $t_{1}$ the distance between *i* and *j*: $d_{ij,t_{1}}$ is given by (2) and (3). $\Delta_{d}ij,t_{1}-t_{0}$ is distance changing of *i*,*j* during $\left[t_{0}-t_{1}\right]$, $v_{j}\left(t\right)$, $v_{i}\left(t\right)$ is the function of velocity.

(5) $$d_{ij,t_{0}}=\sqrt[{2}]{{\left(x_{i,t0}-x_{j,t0}\right)}^{2}+{\left(y_{i,to}-y_{j,t0}\right)}^{2}}$$

(6) $$d_{ij,t_{1}}=d_{ij,t_{0}}+\Delta d_{ij,t_{1}-t_{0}}$$

(7) $$\Delta d_{ij,t_{1}-t_{0}}=\int_{t_{0}}^{t_{1}}\left[v_{j}\left(t\right)-v_{i}\left(t\right)\right]dt.$$

In this vehicle movement model, the link lifetime is defined as the time that the vehicle can maintain communication [30]. Reference [31] proposes link reliability, which is the probability that a direct communication link between two vehicles will stay continuously available over a specified time period. Link reliability can be derived as:

(8) r(l)=P{tocontinuebeavailavlet+Δt∣availableatt}.

**Figure 5:**
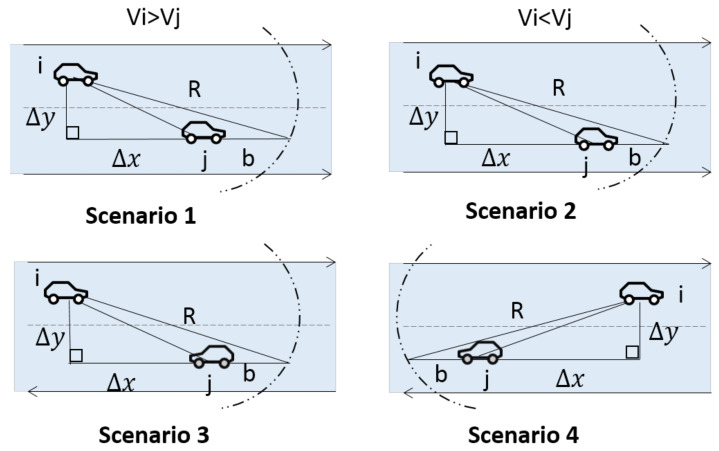
Link stability scenarios.

Next, the vehicle movement will be discussed in several situations in Figure 5:

Scenario 1: Vi and Vj have the same direction, and $v_{i}\geq or\geq v_{j}$. $\Delta d_{ij,t1-t0}$ is the changing distance between node *i* and *j* in the period of $\left[t_{0}-t_{1}\right]$. Δx is the distance on the x-axis, Δy is the distance on the y-axis. *R* is the range of vehicle wireless communication. In this scenario, r(l) can be formulated as:

(9) $$r\left(l\right)=P\left\{\Delta d_{ij,t1-t0}<\sqrt{R^{2}-\Delta y^{2}}+\Delta x\right\}$$

Scenario 2: $v_{i}$ and $v_{j}$ have the same direction, and $v_{i}\leq \leq v_{j}$.

(10) $$r\left(l\right)=P\left\{\Delta d_{ij,t1-t0}<\sqrt{R^{2}-\Delta y^{2}}-\Delta x\right\}.$$

Scenario 3: $v_{i}$ and $v_{j}$ have the opposite direction, and move towards each other, r(l) can be formulated as:

(11) $$r\left(l\right)=P\left\{\Delta d_{ij,t1-t0}<\sqrt{R^{2}-\Delta y^{2}}+\Delta x\right\}.$$

Scenario 4: $v_{i}$ and $v_{j}$ have the opposite direction, move away from each other, r(l) can be formulated as:

(12) $$r\left(l\right)=P\left\{\Delta d_{ij,t1-t0}<\sqrt{R^{2}-\Delta y^{2}}-\Delta x\right\}.$$

The speed and direction of the vehicle can be periodically broadcast to neighbor nodes using hello packets. However, the vehicle speeds $v_{i}$ and $v_{j}$ of the vehicle in $\left[t_{0}-t_{1}\right]$ time can also change. Therefore, the distance changing of *i*, *j* during $\left[t_{0}-t_{1}\right]$$\Delta d_{(}ij,t1-t0)$ can be expressed as:

(13) $$\Delta d_{ij,t1-t0}=\int_{t_{0}}^{t_{1}}\left[v_{j,t0}-v_{i,t0}+\Delta v_{ij}\left(t\right)\right]dt$$

(14) $$\Delta v_{ij}\left(t\right)=\Delta v_{j}\left(t\right)-\Delta v_{i}\left(t\right).$$

In (14), $\Delta v_{ij}\left(t\right)$ is a function of the speed changing between two vehicles with time *t*. The previous study [31], have proved that $\Delta v_{ij}\left(t\right),\Delta v_{i}\left(t\right)$ and $\Delta v_{i}j\left(t\right)$ are all independent variables and follow zero-mean Gaussian distribution. Moreover, the link reliability can be calculated as:

(15) $$r\left(l\right)=\int_{-\infty }^{\frac{D_{i,j}}{t_{1}-t_{0}}\left(v_{j}\left(t_{0}\right)-v_{i}\left(t_{0}\right)\right)}f\left(\Delta v_{j}-\Delta v_{i}\right)\;d\;\left(\Delta v_{j}-\Delta v_{i}\right).$$

where $\Delta v_{j}∼N\left(0,\sigma_{j}^{2}\Delta t\right),\Delta v_{i}∼N\left(0,\sigma_{i}^{2}\Delta t\right)$, and $\Delta v_{j}-\Delta v_{i}∼N\left(0,\sigma_{i}^{2}\Delta t+\sigma_{j}^{2}\Delta t\right)$, so $f\left(\Delta v_{j}-\Delta v_{i}\right)$ is the probability density function (PDF) of Gaussian distribution. $D_{i,j}$ is the range of $\Delta d_{ij,t1-t0}$, which has been discussed in four scenarios.

When the transmission distance is the same, we usually choose the node with good link stability to take the next hop. In fact, the link stability is different and the transmission distance is also different. In order to reduce the routing hops and consider the communication quality, we define the link utility as follows:

(16) $$E\left(l\right)==\frac{d_{ij,t0}}{R}\times r\left(l\right),$$

where $d_{ij,t0}$ is the distance between *i*,*j* at $t_{0}$, *R* is the maximum transmission distance of the node, and r(l) is the link reliability of *i*,*j*.

### 4.2. Vehicle Utility

In order to ensure the minimum number of node hops and increase the connectivity of the link, we consider both link utility and effective information traffic when establishing a route. Before the node forwards the packets, current node has received the hello packet sent by the one-hop neighbors which contains the velocity, direction, position and effective information traffic. The computer can use this information to calculate vehicle utility which is used to select relay node.

When calculating the utilities of the neighbors, we hope that the node with the highest node utility is the highest E(l) and the highest I(t), the vehicle that the utility is the second-highest has the sceond E(l) and I(t), and so on.

**Figure 6:**
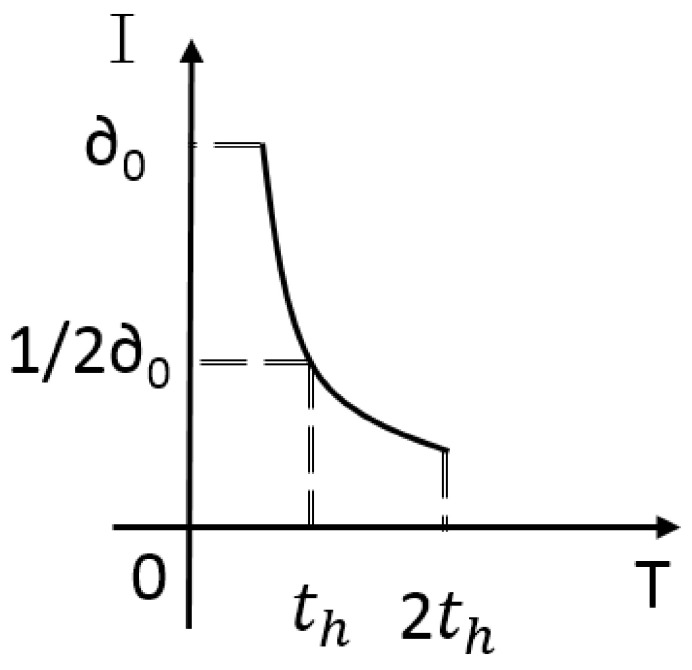
Attenuation function.

The actual situation is often contrary to the ideal state. Usually, the neighbor nodes perform well in one aspect and mediocre on the other. For example, in Figure 6 (two parameters had normalized), each nodes can be expresed by E(l) and I(t),$\left\{E(l),I(t)\right\}$.

**Figure 7:**
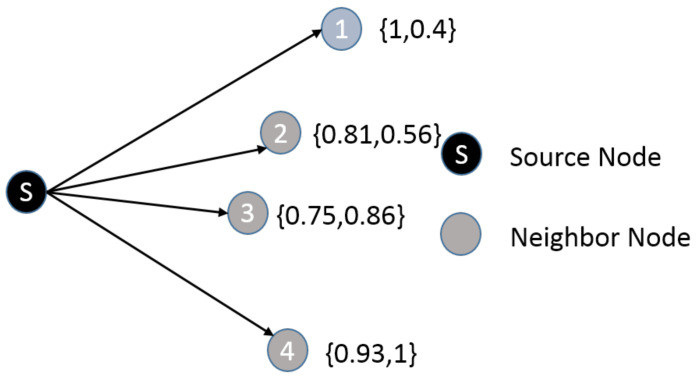
Two parameters of neighbors.

As shown in Figure 7, node 1 is the highest of E(l) but the minimum of I(t). Therefore, node utilities of the neighbors are required to reflect the impact of two parameters on routing. Actually, we cannot compare the effects of these two parameters quantitatively. We use other methods to solve this problem. The parameter whose variance is large has a greater effect on the routing performance than the parameter whose variance is small. In Table 1, the variance of I(t) is lower than that of E(l). Therefore, in the selection process of relaying vehicles, the I(t) should be more effective than the E(l) to affect the routing performance.

Since there are two parameters related to routing performance, in this paper, multi-attribute utility theory (MAUT) [32] is used in our weight calculation method. The weights represent the influence of the parameters on the routing performance. Since parameters with larger variances have greater impact on routing performance than parameters with smaller variances, we will use the variance of the normalized parameters E(l) and I(t) as the weight for calculating the utility of the vehicle. The vehicle utility is defined as follows:

(17) $$U_{i}=E{\left(l\right)}_{i}\times V_{Ei}+I{\left(t\right)}_{i}\times V_{Ii}.$$

where $E{\left(l\right)}_{i}$ is the value of E(l) for node *i*, $V_{Ei}$ is the variance of the E(l); $I{\left(t\right)}_{i}$ is the value of I(t) which is normalized by the maximum value of I(t) in the neighbor table, and $V_{Ii}$ is the variance of $I{\left(t\right)}_{i}$. Before selecting the next hop node, the sender will calculate the $U_{i}$ value for all neighbors. The routing process is described thoroughly in the next subsection.

### 4.3. Routing Process

The real-time effective information traffic routing(RTEIT) is a new vehicle-to-vehicle routing protocol we proposed, which aims to improve communication performance in a sparse urban routing environment. To achieve this goal, we propose a new method to evaluate relay nodes, which has been discussed in other parts of Section 4. In this subsection, we will describe the implementation process of this scheme.

All vehicles broadcast hello massage periodically to neighbor nodes. The receiving node will update the neighbor table according to the information in the hello packet. Each neighbor node has an entry in the neighbor table. Each entry consists of node IP address, speed vector, location coordinates, the value of I(t), and the last update time. This information is directly obtained from the hello package.

**Figure 8:**
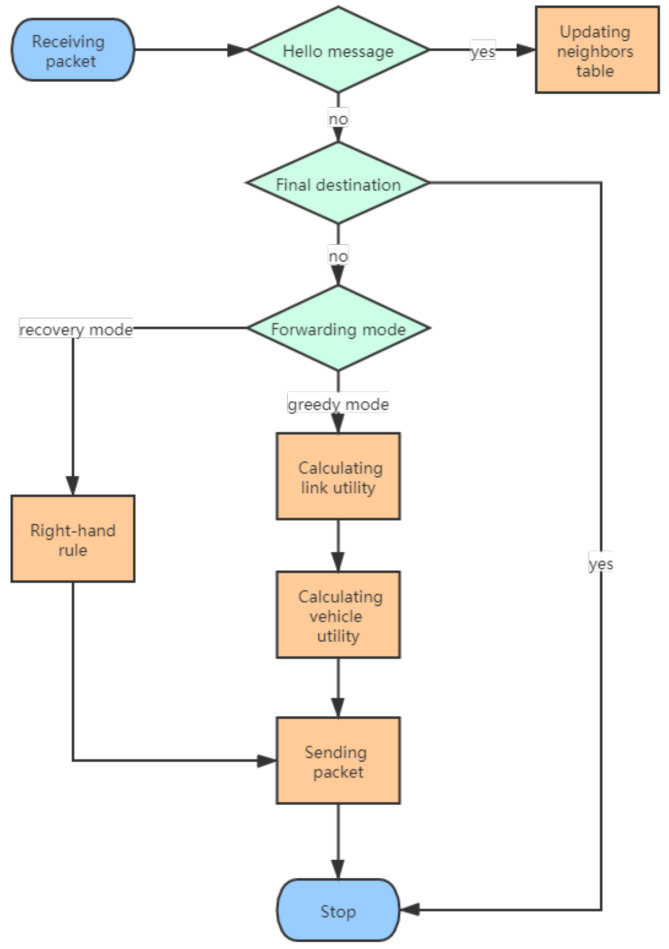
Routing process.

Our new routing scheme is divided into two parts: greedy forwarding and recovery forwarding. Figure 8 shows the whole process of the routing. In greedy forwarding, the source node sends the data packet to the node with the largest vehicle utility from the one-hop neighbor table. In recovery mode, nodes forward packet based on right-hand rule. When the sender wants to send packets, it determines the forwarding mode first according to the location information of the destination node. If there is a neighbor node closer to the destination node, the sender enters a greedy forwarding mode. In another case: the sender has the shortest distance to the destination among neighbors. Generally, there are two kinds of strategies for this condition: one is to carry the packet until it meets the appropriate neighbor which is closer to the destination; the other is to use left or right-hand rule to find nodes that can enter greedy mode. We use the second strategy as the recovery forwarding mode. This is because our protocol is mainly for sparse scenes. In such a scenario, it may take a long time for a node to encounter the next vehicle, which makes the end-to-end delay increase. Another reason is that our defined real-time effective information traffic will reduce the forwarding priority of nodes near the topology hole. In addition, the peripheral forwarding will have a negative impact on the real-time effective information traffic, further reducing the priority of nodes near the road with holes. The details of the algorithm are described in Algorithm 1.
**Algorithm 1** Real-Time Effective Information Traffic Routing (RTEIT) Algorithm**Notations:**CN: Current node. DN: Destination node. *P*: Packet. $N_{T}$: Neighbor table. $d_{n}eighbor$: Distance between neighbor and destination. $d_{c}$: Distance between current node and destination.1:**while** (CN receive *P*) **do**2:    **if** *P* is hello packet **then**3:        $n_{p}$=get position from hello packet;4:        $n_{v}$=get speed from hello packet;5:        $n_{i}$=get I(t) from hello packet;6:        add $n_{p}$,$n_{v}$,$n_{i}$ to $N_{T}$ or refresh it;7:    **end if**8:    **if** *P* is data packet **then**9:        **if** in greedy mode **then**10:           calculate $d_{c}$;11:           **for** (each $neighbor\in N_{T}$) **do**12:               calculate $d_{n}eighbor$;13:               **if** $d_{neighbor}>d_{\min}$ **then**14:                   $d_{neighbor}\leftarrow d_{\min}$;15:               **end if**16:           **end for**17:           **if** $d_{c}<d_{\min}$ **then**18:               enter to recovery mode;19:           **else**20:               **for** (each $neighbor\in N_{T}$) **do**21:                   calculate E(l) for neighbor;22:               **end for**23:               calculate $U_{neighbor}$ for each neihgbor;24:               **for** (each $neighbor\in N_{T}$) **do**25:                   **if** $U_{max}<U_{neighbor}$ **then**26:                       $U_{max}\leftarrow U_{neighbor}$27:                       bestnode=neighbor;28:                   **end if**29:               **end for**30:               forward to bestnode in greedy mode;31:           **end if**32:        **else**33:           forward in recovery mode;34:        **end if**35:    **end if**36:**end while**

### 4.4. Complexity Analysis

In this section, we will investigate the complexity of Algorithm 1. We assume that the number of neighbors of the sender is n. In Algorithm 1 the sender needs to calculate the distance of each node to the destination firstly for forwarding mode selection. The time complexity of this process is O(*n*). In the greedy forwarding mode, according to Formula (16), additional calculation of link utility E(l) is required. As shown in Formula (15), E(l) is defined as an integral formula, where $f(\Delta v_{j}-\Delta v_{i})$ follows Gaussian distribution. Therefore, the complexity of (15) is O(1) using Newton–Leibniz formula to the computation, and for all neighbors the complexity is O(*n*). Due to the effective information flow is maintained by each node and sent to the neighbors with hello packet, the complexity of I(t) is O(1). As shown in Formula (16), in order to obtain the value of vehicle utility, it is necessary to calculate the variance of E(l) and I(t), so the computation complexity is O(1). In general, the computing complexity of each node in greedy forwarding mode is O (*n*), and the complexity of the whole routing process is O ($n^{2}$). In the recovery mode, the right-hand rule is the core of the algorithm. A no-crossing algorithm based on RNG or GG network structure is used in this mode, which requires O($n^{2}$) complexity for each node. For the whole routing path the complexity is O($n^{3}$). As shown in the Table 2, compared with the classical routing protocol, our protocol does not increase the complexity.

## 5. Simulation and Results

In this section, we evaluate the RTEIT protocol proposed in this paper through NS-3 simulator. SUMO provides tracefile as the simulation mobile scenario. GPSR is the most classic location-based routing, and its results are used as our comparison group. MM-GPSR is an improvement of GPSR, which improves the link quality by limiting the range of *Q*. In our proposed scheme, link quality is considered and redundant paths are reduced by new parameters. The results of RTEIT are compared with GPSR, MOPR, and MM-GPSR.

### 5.1. Network Configuration

**Figure 9:**
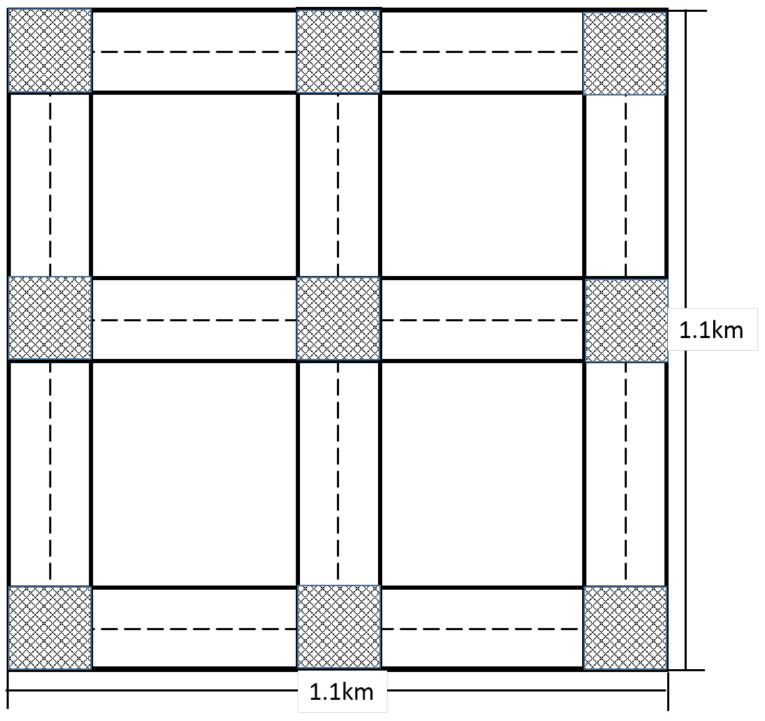
Simulation area.

As shown in Figure 9, there are 12 streets and nine intersections in the simulation area. The nodes in this area are placed randomly at the beginning, and they move to a random destination with a speed not exceeding 15 m/s.

The simulation experiments were implemented on ns-3.27. For sparse traffic scenarios, the number of vehicles is set to 30, 40, 50, 60, 70 and nodes with a maximum node speed of 15 m/s. The maximum communication radius of the vehicle was set to 250 m. Accurate vehicle position information can be provided by the positioning system, in other words, the error of GPS is not considered. The data traffic is considered as Constant Bit Rate(CBR). And the experiment of each scene was run for 150 s. Other parameter values are assigned on the basis of the values used in [28]. Table 3 summarizes the key parameters in the simulation.

### 5.2. Performance Metrics

We evaluate the performance of routing protocols using the following performance metrics.

1. Packet loss rate: This represents the ratio of the number of data packets that are lost to the total number of data packets sent at the source node.
  (18) $$\mathrm{Packet}\;\mathrm{loss}\;\mathrm{rate}=\frac{P_{l}}{P_{s\;}}\times 100$$
2. End-to-end delay: This represents the average delay of packets that are generated at the source node and received successfully at the destination.
  (19) $$\mathrm{End}-\mathrm{to}-\mathrm{end}\;\mathrm{delay}=\frac{\sum_{n=1}^{N}D_{n}}{N}$$
3. Network yield: This represents the comprehensive performance of the network. Defined as the ratio of the total packets received at the destination to the total number of packets sent by all nodes of the network.
  (20) $$\mathrm{Network}\;\mathrm{yield}=\frac{P_{r}}{P_{all}}$$

### 5.3. Packet Loss Rate

**Figure 10:**
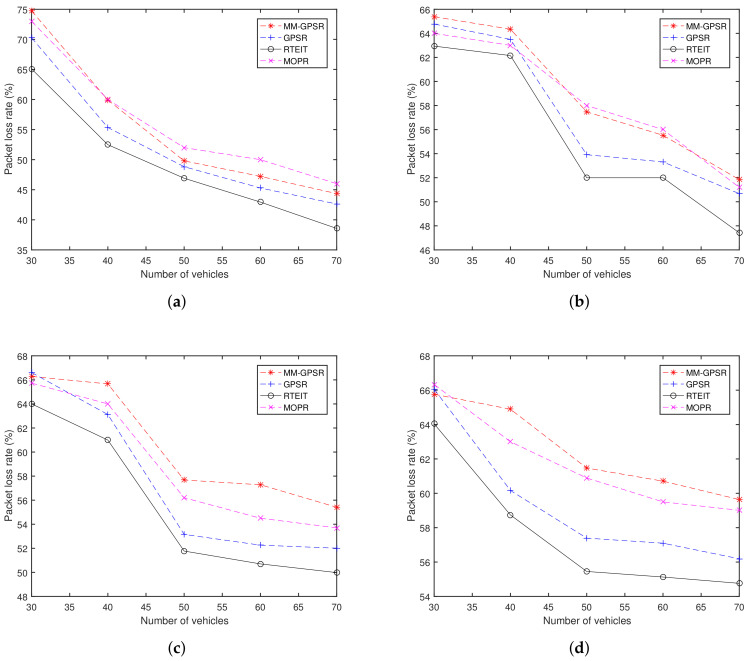
Packet loss rate for varying the number of vehicles and CBR connections: (**a**) 5 CBR connections; (**b**) 10 CBR connections; (**c**) 15 CBR connections; (**d**) 20 CBR connections.

Figure 10 shows the packet loss rate for varying the number of vehicles and CBR connections. In macroscopical sight, when the number of nodes is increasing, the packet loss rates are reduced in all protocols. This occurs due to the increase in node density can improve the network connectivity and reduce network partition, which increases the probability that a packet successfully reaches the destination node. In the sparse environment, where the number of nodes from 30 to 70, data packets are more likely to be discarded in the process of finding the correct path. In general, RETIT has a lower packet loss rate than MM-GPSR, MOPR, and GPSR in sparse scenario.

For instance, as Figure 10a shows, in the scenario with 5 CBR connections, all the RETIT points have a lower packet loss rate than MOPR, MM-GPSR, and GPSR. This can be explained as that neither GPSR nor MM-GPSR considers link quality during the process of making decisions. RETIT and MOPR can avoid link disruption. MM-GPSR selects the next hop, which is not the nearest to the destination, resulting in an increase in the number of hops which can improve the probability of a packet dropped by vehicles. Compared with the results in Figure 10b, these three protocols have an increase in packet loss rate at 70 nodes. Such a phenomenon can be explained as: when the number of CBR connection pairs increases, the network load increases, and the chances of collision and interference between packets increases, resulting in poor performance of packet loss rate. Another reason why RETIT performs better than GPSR and MM-GPSR on packet loss rate is that RETIT selects the correct path. We assume that the probability of packet loss per hop is equal, then more hops mean more packets are discarded. Real-time effective information traffic mechanism reduces the occurrence of redundant paths and further reduces the packet loss rate. For packet loss rate, the TREIT performance 5% better than GPSR, 8% better than MM-GPSR, and 7% better than MOPR. On the whole, the packet loss rate of RTEIT is smaller than GPSR, MOPR, and MM-GPSR in the sparse scenario, that is because RTEIT selects the next hop with a more stable connection, smaller distance to destination, and better connectivity.

### 5.4. End-to-End Delay

**Figure 11:**
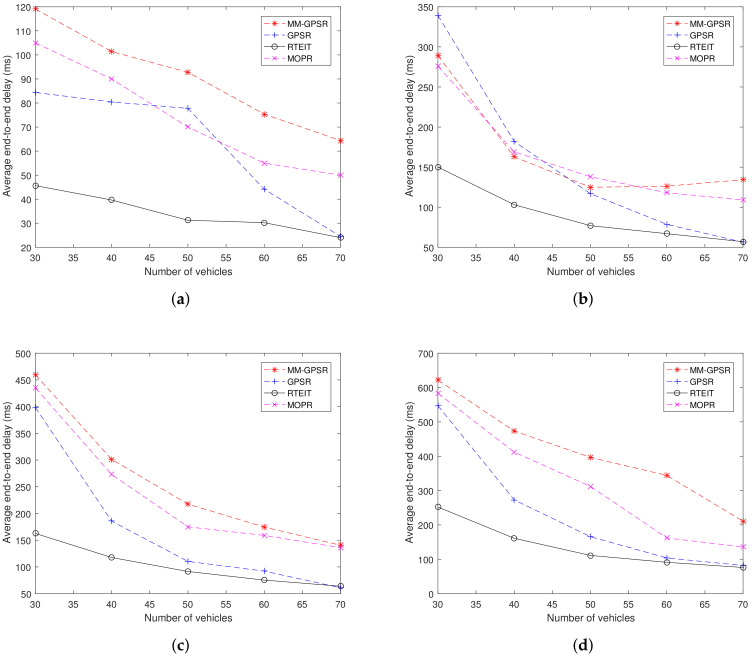
End-to-end delay for varying the number of vehicles and CBR connections: (**a**) 5 CBR connections; (**b**) 10 CBR connections; (**c**) 15 CBR connections; (**d**) 20 CBR connections.

Figure 11 shows the end-to-end delay for varying the number of vehicles and CBR connections. It can be noted from Figure 11 that as the number of nodes increases, the performance of end-to-end delay becomes better in three protocols. Compared with GPSR, MOPR, and MM-GPSR, RTEIT obviously has smaller end-to-end latency. For instance, the end-to-end delay of RTEIT is about 20% lower than GPSR, 40% lower than MM-GPSR, and 30% than MOPR.In the process of successful transmission, the end-to-end delay is mainly determined by the number of hops. When the vehicle nodes are sparse, the packets frequently enter recovery forwarding mode which generates more hops in the routing. That can explain why the performance of end-to-end delay at 70 vehicles is clearly better than results at 30 vehicles. For GPSR, MOPR, and MM-GPSR, due to the lack of adequate consideration about network environment, data packets are likely to enter sparse road segment where the connection between nodes is not sufficient and more likely to encounter topological holes. For RTEIT, the establishment of routing is determined by real-time information traffic and vehicle movement. Due to the poor connectivity, the nodes around the topology hole frequently enter the recovery mode and have lower real-time information traffic in the calculation of vehicle utility value. Nodes with more available connections have a higher priority in routing. By choosing the next hop with high connectivity, RTEIT can minimize the use of recovery strategy, resulting in lower end-to-end delay than GPSR, MOPR and MM-GPSR. In most cases, GPSR outperforms the MM-GPSR because MM-GPSR provides a scheme that uses a fixed transport factor λ to determine the communication area Q, thus the value of λ is not suitable for the scenarios used in the experiment. From Figure 11d, the value of maximum end-to-end delay for MM-GPSR is 620 ms, for GPSR is 560 ms, and for RETIT is 240 ms. The values of minimum end-to-end delay for these protocols are 280, 120, 170, and 100. The gap between traditional protocols and RETIT on end-to-end delay reduces with the increase in the number of nodes. This clearly demonstrates that RETIT has superiority compared with MM-GPSR, MOPR, and GPSR. In Figure 11b, it can be found that MM-GPSR outperforms GPSR at 30 and 40 vehicles. This result can be interpreted as: the benefits of fixed parameters λ equal to 0.3. From Figure 11, it also can be found that with the increase in the number of CBR connections, the end-to-end delay increases. More CBR connections lead to more serious network load, which prevents the nodes from forwarding the data packet just received because the packets in the queue have higher priority.

### 5.5. Network Yield

**Figure 12:**
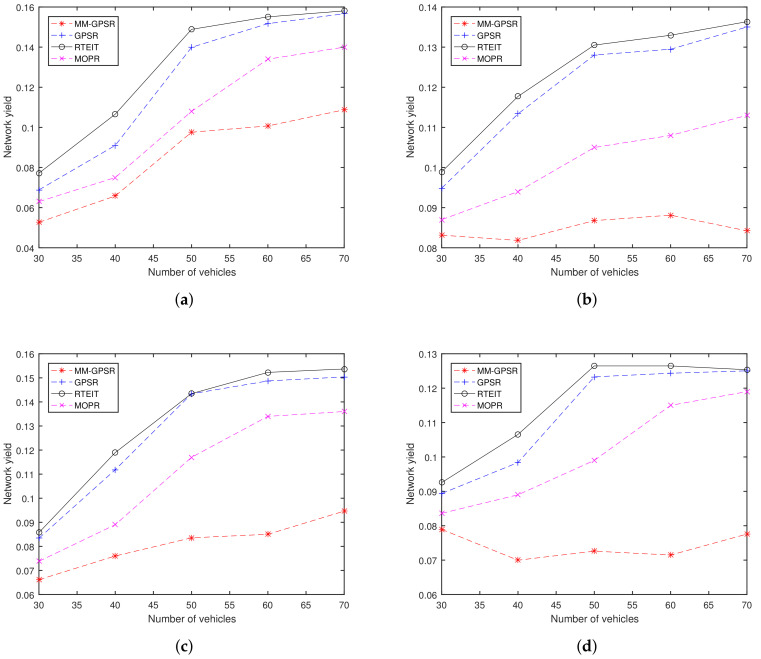
Network Yield for varying the number of vehicles and CBR connections: (**a**) 5 CBR connections; (**b**) 10 CBR connections; (**c**) 15 CBR connections; (**d**) 20 CBR connections.

Figure 12 shows the network yield for varying the number of vehicles and CBR connections. The network yield represents the comprehensive performance of a network, which similar to the goodput. For example, when the packet loss rate increases, the number of packets that reach the destination becomes smaller, thus, the value of network yield is small. In the same condition of packet loss rate, a route with a large number of hops will have a negative impact on network yield. From Figure 12, it can be found that when the number of nodes increases, the performance of network yield is better (with exception of Figure 12d, where the number of CBR connections is 20, the results of MM-GPSR are similar). The main reason to limit the performance in a sparse network is that the network connection is not enough due to the small number of nodes. Actually, location-based greedy forwarding mode is very suitable for well-connected networks due to this scheme aims to obtain the minimum number of hops. From Figure 10 and Figure 11, we can found that RETIT has a lower packet loss rate and lower end-to-end delay. Obviously, our proposed RTEIT has a higher network yield in a sparse environment by comparing with GPSR, MOPR, and MM-GPSR in all set of CBR connections. These results can be interpreted as: (1) link utility considers both one-hop transmission distance and link stability, which increases the packet delivery ratio and reduces the number of hops; (2) effective information traffic leads nodes to find a correct path to a destination which can avoid path redundancy.

## 6. Conclusions

In this paper, we present a location-based routing algorithm named RTEIT that provides a new method of selecting the next hop taking advantage of link stability, distance and effective information traffic for choosing the most appropriate relay node. The link utility is used to assess the link status which is based on the distance and stability of the wireless transmission. The effective information traffic mechanism is used to lead a correct path. The experiment of the proposed algorithm is successfully established in a spare scenario in NS-3. Simulation results indicate that RTEIT outperforms both GPSR, MOPR, and MM-GPSR in term of packet loss rate, end-to-end delay and network yield. Our work assumes that global positioning system can provide accurate location information. However, due to the multipath effect and other factors, geographic location information is generally inaccurate, which will make degradation of protocol performance in VANETs [33]. Therefore, in future work, we will consider this situation and make corresponding improvements to adapt to the actual scene.

## Figures and Tables

**Table 1:** Parameters with diferent variances.

	Node 1	Node 2	Node 3	Node 4	Variance
I(t)	0.4	0.56	0.86	1	0.075
E(l)	1	0.81	0.75	0.93	0.012

**Table 2:** Complexity comparison of GPSR and real-time effective information traffic routing (RTEIT).

	GPSR	RTEIT
Greedy forwarding	$n^{2}$	$n^{2}$
Recovery forwarding	$n^{3}$	$n^{3}$

**Table 3:** Simulation parameter.

	Simulation Parameter	Value
	Simulation area	1100 m × 1100 m
	Number of streets	12
	Number of vehicles	30, 40, 50, 60, 70
	Transmission range	250 m
	Packet size	512 byte
	Simultaion time	200 s
	MAC layer	IEEE 802.11p
	Simulation tool	Ns3
	Number of CBR connections	5, 10, 15, 20
	Max speed	15 m/s
	Propagation model	Two-ray ground
	Beacon Interval	1 s
